# Alliinase from *Ensifer adhaerens *and Its Use for Generation of Fungicidal Activity

**DOI:** 10.1186/2191-0855-1-2

**Published:** 2011-03-28

**Authors:** Masahiro Yutani, Hiroko Taniguchi, Hasibagan Borjihan, Akira Ogita, Ken-ichi Fujita, Toshio Tanaka

**Affiliations:** 1 Department of Biology and Geosciences, Graduate School of Science, Osaka City University, 3-3-138 Sugimoto, Sumiyoshi-ku, Osaka 558-8585, Japan; 2 Research Center for Urban Health and Sports, Osaka City University, 3-3-138 Sugimoto, Sumiyoshi-ku, Osaka 558-8585, Japan

## Abstract

A bacterium *Ensifer adhaerens *FERM P-19486 with the ability of alliinase production was isolated from a soil sample. The enzyme was purified for characterization of its general properties and evaluation of its application in on-site production of allicin-dependent fungicidal activity. The bacterial alliinase was purified 300-fold from a cell-free extract, giving rise to a homogenous protein band on polyacrylamide gel electrophoresis. The bacterial alliinase (96 kDa) consisted of two identical subunits (48 kDa), and was most active at 60°C and at pH 8.0. The enzyme stoichiometrically converted (-)-alliin ((-)-*S*-allyl-L-cysteine sulfoxide) to form allicin, pyruvic acid, and ammonia more selectively than (+)-alliin, a naturally occurring substrate for plant alliinase ever known. The C-S lyase activity was also detected with this bacterial enzyme when *S*-alkyl-L-cysteine was used as a substrate, though such a lyase activity is absolutely absent in alliinase of plant origin. The enzyme generated a fungicidal activity against *Saccharomyces cerevisiae *in a time- and a dose-dependent fashion using alliin as a stable precursor. Alliinase of *Ensifer adhaerens *FERM P-19486 is the enzyme with a novel type of substrate specificity, and thus considered to be beneficial when used in combination with garlic enzyme with respect to absolute conversion of (±)-alliin to allicin.

## Introduction

**Figure 1:**
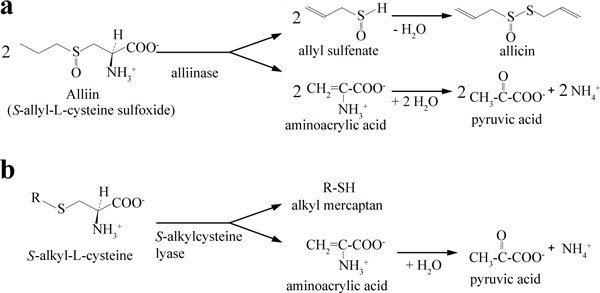
**Reactions of alliinase (a) and *S*-alkylcyteine lyase (b)**.

Allicin (diallyl thiosulfinate, Figure 1a) is the best-known active compound of freshly crushed garlic extract, and is known to possess a vast variety of biological effects: antimicrobial, anti-inflammatory, antithrombotic, anticancer, and antiatherosclerotic activities (Stoll and Seeback 1951; Block 1985; Tsai et al. 1985; Agarwal 1996; Ankri and Mirelman 1999; Siegel et al. 1999). This allyl-sulfur compound is synthesized as a result of condensation of allyl sulfenate, which is produced depending on the C-S lyase activity of alliinase (EC 4.4.1.4) on (+)-alliin ((+)-*S*-allyl-L-cysteine sulfoxide), a naturally occurring diastereomer, as illustrated in Figure 1a (Siegel et al. 1999; Jones et al. 2004). Alliinase is therefore distinguished from *S*-alkyl-L-cysteine lyase (EC 4.4.1.6), which simply exhibits C-S lyase activity as to produce alkyl mercaptan in addition to pryruvic acid and ammonia (Figure 1b). Alliinase has been purified from garlic, onion, and other plants of the genus *Allium *(Schwimmer and Mazelis 1963; Mazelis and Crews 1968; Tobkin and Mazelis 1979; Nock and Mazelis 1986; Landshuter et al. 1994; Lohmüller et al. 1994; Rabinkov et al. 1994; Manabe et al. 1998). The enzymatic production of allicin is thought to occur in nature as a result of injury of the plant tissue that enables interaction of the enzyme in vacuoles with alliin accumulated in the cytosol (Lancaster and Collin 1981). Therefore, allicin production has been discussed as a defense mechanism of the plant against microbial infection or insect attack (Slusarenko et al. 2008).

On-site production of allicin from a stable precursor alliin is attractive if this can be applied for various clinical purposes. In fact, with increasing interest in the efficacy of allicin in the purposes, its production has been devised both *in vitro *and *in vivo *with the aid of garlic alliinase (Miron et al. 2003; Shadkchan et al. 2004; Fry et al. 2005). Alliinase from microbial origin is another choice for this purpose. (Murakami (1960)) first reported the occurrence of alliinase in microbial world based on the detection of the corresponding enzyme activity in acetone-powdered cell-free extract from *Bacillus subtilis*. (Durbin and Uchytil (1971)) then reported the production of the enzyme by *Penicillium corymbiferum*. The partially purified enzyme stoichiometrically converted alliin to pyruvic acid, ammonia, and allicin, showing none of lyase activity on *S*-alkyl-L-cysteine. In the present study, we isolated a microorganism, which generates allicin-like odor during the growth in medium containing (±)-alliin, and purified the enzyme involved in the metabolic conversion of this amino acid derivative into an odorous compound. We hereby report isolation of an alliinase-producing bacterium, purification of the bacterial enzyme, and its substrate specificity characterized by the selectivity toward (-)-alliin. We also evaluate a combination of the enzyme and alliin with respect to on-site generation of allicin-dependent fungicidal activity using the yeast *Saccharomyces cerevisiae *as a model of fungal cells.

## Materials and methods

### Isolation of alliinase-producing microorganism

Appropriately diluted suspension of each soil sample was plated onto a synthetic medium (12% (*w/v*) Na_2_HPO_4_, 0.3% KH_2_PO_4_, 0.1% D-glucose, 0.05% NaCl, 0.002% CaCl_2_, 0.0003% MgSO_4_·7H_2_O, and 2% (*w/v*) agar, pH 6.2), in which (±)-alliin was added at the concentration of 0.1% as a sole nitrogen source. After 7-days incubation at 25°C, colonies formed were isolated as alliin-utilizing strains, and were independently inoculated onto the agar plates for evaluation of odorous compound production. Six strains were chosen, but cells of these strains were poorly grown in the liquid synthetic medium with the above composition. Therefore, alliinase activity was assayed with cells cultivated in the nutrient medium as described in the following section. A strain with the highest activity was chosen and designated strain FERM P-19486, which has been deposited in the National Institute of Advanced Industrial Science and Technology (Tsukuba, Japan).

### Identification of isolated strain FERM P-19486

Strain FERM P-19486 was identified on the basis of 16S rDNA sequence in addition to the morphological and physiological properties examined according to the guideline of (Casida (1982)). The genomic DNA was extracted with the DNeasy Tissue Kit (QIAGEN, Valencia, CA). PCR amplification of the 16S rDNA and its sequence analysis were performed with the MicroSeq Full Gene 16S rDNA kit (Applied Biosystems, Foster City, CA).

### Enzyme assay

The standard alliinase assay mixture contained 40 mM (±)-alliin, 20 μM pyridoxal 5'-phosphate (PLP), 50 mM sodium phosphate (pH 7.0), and enzyme in a total volume of 1.0 ml. Alliinase activity was determined by colorimetrically measuring pyruvic acid produced in the reaction as described by (Durbin and Uchytil (1971)). One unit of the enzyme activity was defined as the enzyme amount that catalyzed the formation of 1 μmole of pyruvic acid per min at 30°C. Protein concentration was measured according to the method of Bradford using bovine serum albumin as a standard (Bradford, 1976).

### Measurement of alliin, allicin, and ammonia

In the reaction with (±)-alliin, the remaining concentrations of (+)-alliin and (-)-alliin were measured by HPLC using a reverse-phase C_18 _column (YMC-Pack ODS-AM, YMC Co., Kyoto, Japan) at 220 nm. In this HPLC analysis, (+)-alliin and (-)-alliin could be separated by isocratic elution, which was done with 10 mM sodium phosphate buffer (pH 7.5) containing 5 mM tetra *n*-butylammonium dihydrogenphosphate at a flow rate of 1.0 ml per min. Allicin was also measured by HPLC using the same column except that it was isocratically eluted with a mixture of acetonitrile, H_2_O, and tetrahydrofuran at a ratio of 30: 67: 3 (*v/v*) and detected at 240 nm. Ammonia were colorimetrically measured as described by (Mazelis and Creveling (1975)).

### Enzyme purification

#### (*i*) *Preparation of cell free extract*

Strain FERM P-19486 was routinely grown in the nutrient medium, which consisted of 3% (*w/v*) bouillon (Nissui Co., Tokyo, Japan), at 30°C for 3 d with vigorous shaking. Cells from 1,000 ml culture were collected by centrifugation at 7,000 × *g *for 10 min, washed, and suspended with 0.02 M sodium phosphate buffer, pH 7.0 (buffer A). Cells were then disrupted by ultrasonic treatment at 0°C using a Branson Sonifier 250, and the supernatant obtained after removing cell debris was used as a crude enzyme.

#### (*ii*) *DEAE-cellulose column chromatography*

The supernatant was put on a DEAE-cellulose column (3.0 × 14.0 cm) equilibrated with buffer A. After washing the column with the same buffer, the enzyme was eluted with buffer A containing 0.05 M NaCl.

#### (*iii*) *Phenyl-sepharose column chromatography*

After addition of NaCl to the active fraction at 1.5 M, it was then put on a phenyl-sepharose (Amersham Pharmacia Biotech, Uppsala, Sweden) column (1.5 × 3.0 cm) equilibrated with buffer A containing 1.5 M NaCl. After washing the column with the same buffer, the enzyme was eluted with buffer A. The active fractions were combined and dialyzed against buffer A.

#### (*iv*) *Aminohexyl-sepharose column chromatography*

The enzyme was put on an aminohexyl-sepharose (Sigma-Aldrich, St. Louis, MO) column (1.5 × 6.0 cm) equilibrated with buffer A. After the column was washed with the same buffer, the enzyme was eluted with a linear gradient of buffer A to buffer A containing 0.3 M NaCl. The volume of each fraction was 5 ml. The active fractions were combined and concentrated to about 500 μl with an Ultrafree-MC (30,000 NMWL, Millipore, Bedford, MA).

#### (*v*) *Mono Q column chromatography*

The enzyme solution was then applied to a Mono Q (Pharmacia, Uppsala, Sweden) column (5.0 × 50 mm) equilibrated with buffer A. After the column was washed with the same buffer, the enzyme was eluted with a linear gradient of buffer A to buffer A containing 0.2 M NaCl. The flow rate was 1 ml/min. The active fractions were combined and concentrated to about 50 μl with an Ultrafree-MC (30,000 NMWL).

#### (*vi*) *Gel filtration*

The enzyme was then applied to a TSK-GEL (TOSOH, Tokyo, Japan) column (7.8 × 300 mm) equilibrated with buffer A. The flow rate was 1 ml/min. The active fractions were combined and used as the purified enzyme.

### Electrophoresis

The purity of the enzyme was examined by native-polyacrylamide gel electrophoresis (PAGE) using 8% polyacrylamide gel at a constant current of 20 mA per gel at 4°C. For detection of alliinase activity, gel slices were cut from another lane with the same sample, and each gel slice was directly incubated in 100 μl of the standard alliinae assay mixture at 30°C for 30 min. Allicin produced in the mixture was measured by HPLC. Sodium dodecyl sulfate (SDS)-PAGE was carried out using 10% (*w/v*) polyacrylamide gel at a constant current of 20 mA per gel, in which broad-range molecular mass standards (Bio-Rad Laboratories, Tokyo, Japan) were simultaneously run. Proteins were detected by silver staining.

### Molecular mass determination

The molecular mass of the native enzyme was estimated by gel filtration using a TSK-GEL column. The operating condition was already described above. The column was calibrated by using the standard proteins: thyroglobulin (669 kDa), ferritin (440 kDa), catalase (232 kDa), lactate dehydrogenase (140 kDa), and bovine serum albumin (66 kDa). The molecular mass of the enzyme under denaturing conditions was determined by SDS-PAGE.

### Measurement of cell viability in medium containing (±)-alliin and P-19486 alliinase

*S. cerevisiae *W303-1A cells were grown at 30°C for 16 h with vigorous shaking in YPD medium containing 1% (*w/v*) yeast extract, 2% (*w/v*) peptone, and 2% (*w/v*) D-glucose. An overnight-grown culture was diluted with freshly prepared YPD medium to obtain an initial cell density of 10^7 ^cells/ml, in which PLP was supplemented at 20 μM. Incubation was then started at 30°C with the addition of various concentrations of (±)-alliin and FERM P-19486 alliinase. Aliquots of the cell suspension were withdrawn, diluted, and spread onto YPD-agar plates to measure the viable cell number as colony-forming units after a 24-h incubation at 30°C.

### Chemicals

(±)-Alliin, (+)-alliin, and allicin were products of LKT Laboratories, Inc (St. Paul, MN). *S*-Methyl-L-cysteine and *S*-ethyl-L-cysteine were obtained from ICN Pharmaceuticals, Inc (Costa Mesa, CA). *S*-Methyl-L-cysteine sulfoxide was from Research Organics, Inc (Cleveland, OH).

## Results

### Identification of strain FERM P-19486

As summarized in Table 1, strain FERM P-19486 is a rod-shaped bacterium with a size of 1.2-2.0 μm in length and 0.6-0.7 μm in diameter. The bacterium was Gram-negative, aerobic, non-spore forming, motile, catalase-positive, and cytochrome oxidase-positive. Gelatin and starch were not utilized. In addition to these morphological and physiological properties, the carbohydrate and amino acid assimilation patterns were identical to those described for a type culture of *Ensifer adhaerens *except for the growth defect of the isolated strain FERM P-19486 at 37°C (Casida 1982; Sawada et al. 2003). 16S rDNA sequence of strain FERM P-19486 showed 100% homology to that of *E. adhaerens *ATCC 33499 (Rogel et al. 2001), which is sited at GenBank (AF191738, http://www.ncbi.nlm.nih.gov/nuccore/6180038), agreeing with the identification of the strain to be *E. adhaerens*.

**Table 1:** Tanxonomical features of strain FERM P-19486

Shape	Short rods (0.6 - 0.7 × 1.2 - 2.0 μm)
Motility	+
Spore	-
Gram stain	-
Anearobic growth	-
Cytochrome oxidase	+
Catalase	+
OF test	Acid
Nitrate reduction	+
Urease	+
Gelatin liquefaction	-
Starch hydrolysis	-

### Purification of P-19486 alliinase

**Figure 2:**
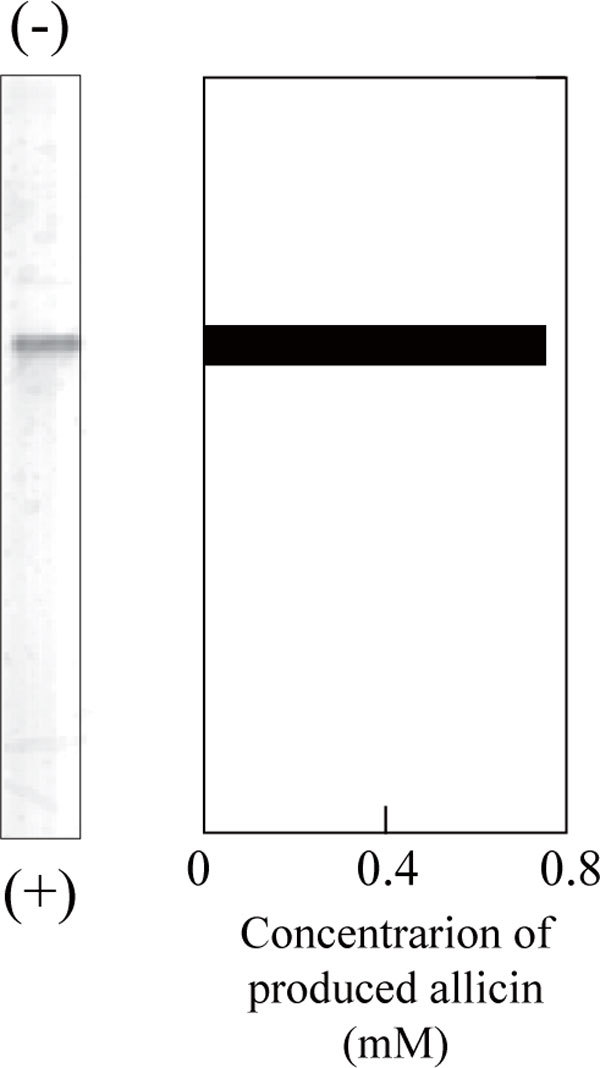
**PAGE of purified P-19486 alliinase (left) and the detection of the enzyme activity on the gel (right)**. The purified enzyme (100 ng) was applied to each lane and was run at a constant current of 20 mA at 0°C. After electrophoresis, one lane was used for protein staining (left) and the other one was for alliinase assay. Closed bar of right hand side indicates the location of allicin production on the gel

The enzyme was purified 300-fold from the crude extract with the overall yield of 1.2% (Table 2). The purified preparation was shown to be homogenous by native-PAGE, in which allicin produced from alliin was detected with the corresponding protein band (Figure 2). The specific activity of P-19486 alliinase was slightly reduced at the final step of purification, suggesting the loss of stability in association with the purity increase. The addition of PLP to buffer A used during chromatography was ineffective in preventing the loss of activity. Unlike the case with heat denaturation, however, the specific activity remained at a constant level for at least 1 week at -20°C or even at 4°C. It remains to be solved whether the enzyme requires a certain factor for the maximum activity.

**Table 2:** Purification of alliinase from *E. adhaerens *FERM P-19486

Purification step	Protein (mg)	Total activity (units)	Sp. Act (units/mg)	Yield (%)	Purification (fold)
Crude extract	209.81	73.0	0.3	100	1
DEAE-cellulose	9.60	30.0	3.1	41.1	10
Phenyl-sepharose	1.94	22.4	11.5	30.7	38
Aminohexyl-sepharose	0.29	7.5	25.9	10.3	86
Mono Q	0.04	4.1	102.5	5.6	341
TSK-GEL	0.01	0.9	90.0	1.2	300

### Physicochemical properties

**Figure 3:**
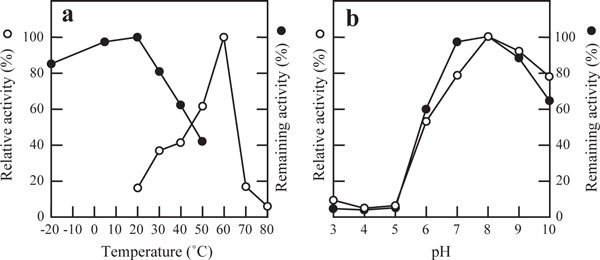
**Effects of temperature (a) and pH (b) on P-19486 alliinase**. In (a), the optimum temperature (open circle) was determined by incubating the enzyme under the standard assay condition at each temperature. In the assay of thermal stability (closed circle), the enzyme activity was measured by the addition of (±)-alliin to the standard assay mixture, in which the enzyme had been preincubated at each temperature for 30 min. In (b), the optimum pH (open circle) was determined by incubating the enzyme under the standard assay condition where pH of the mixture was adjusted as indicated. In the assay of pH stability (closed circle), the enzyme activity was measured by the addition of (±)-alliin to the standard assay mixture, in which the enzyme had been preincubated at 4°C and at each pH for 30 min

P-19486 alliinase had a molecular mass of 96 kDa, and consisted of two subunits identical in molecular mass (48 kDa), as judged by the analyses based on gel filtration and SDS-PAGE. P-19486 alliinase was most active at 60°C, though the enzyme was unstable above 20°C, as seen from the loss of activity to 42% of the maximum value (Figure 3a). The enzyme was stable in the alkaline pH range between 7.0 and 9.0, in which the maximum activity was detected at around pH 8.0 (Figure 3b). This suggested that the substrate alliin contributed to the maintenance of enzyme stability during incubation at 60°C. Alliinases so far reported are typical PLP-dependent enzyme (Tobkin and Mazelis 1979; Kuettner 2002a, b). The activity of P-19486 alliinase in the absence of PLP was only 14.3% of that detected in the presence of 20 μM PLP, suggesting that PLP is also a cofactor of this enzyme.

### Selectivity toward diastereomer of alliin

**Figure 4:**
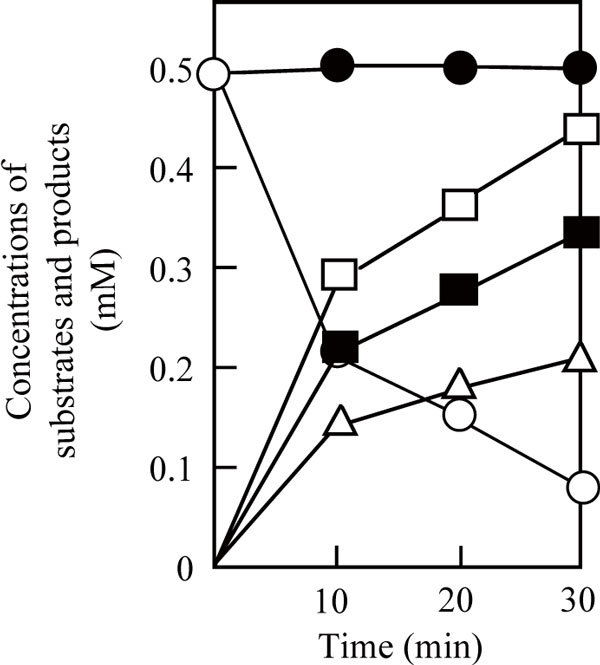
**Conversion of (±)-alliin to pyruvic acid, ammonia, and allicin by P-19486 alliinase**. The reaction mixture containing 1 mM (±)-alliin, 20 μM PLP, and P-19486 alliinase (0.1 unit) in 1.0 ml of 50 mM sodium phosphate buffer (pH 7.0) was incubated at 30°C. Portions were withdrawn for the measurement of pyruvic acid (open square) and ammonia (closed square) by the colorimetric methods. The concentrations of (+)-alliin (closed circle), (-)-alliin (open circle), and allicin (open triangle) were measured by the HPLC-analyses.

Two diastereomers, (+)-*S*-allyl-L-cysteine sulfoxide and (-)-*S*-allyl-L-cysteine sulfoxide, exist in the molecular structure of alliin because of the asymmetry of the sulfoxide group. As summarized in Table 3, P-19486 alliinase exhibited the lyase activity on (±)-alliin more effectively than (+)-alliin, reflecting its selectivity toward (-)-alliin. The enzyme was also active on (±)-*S*-methyl-L-cysteine sulfoxide with a higher *V*_max _value than (+)-alliin. This should also reflect its selective action on (-)-isomer of the substrate. The *K*m value of P-19486 alliinase for (±)-alliin was 2.3 mM, and was apparently lower than that for (+)-alliin (5.7 mM), suggesting that the *K*m value for (-)-alliin might be around 1 mM or less. We therefore examined whether P-19486 alliinase can selectively react with (-)-alliin under the condition where the concentration of each diastereomer was adjusted to the level less than the possible *K*m value for (-)-alliin. As shown in Figure 4, P-19486 alliinase could selectively decompose (-)-alliin, whereas the level of (+)-alliin was kept unchanged at least during 30-min incubation.

**Table 3:** Substrate specificity of alliinase from *E. adhaerens *FERM P-19486

Substrate	Kinetic constants		
	
	*V*_max_^a^ (units/mg)	*K*m (mM)	*V*_max_*/K*m
(±)-Alliin	90.0	2.3	39.1
(+)-Alliin	18.5	5.7	3.2
*S*-Allyl-L-cysteine	66.1	10.6	6.2
*S*-Methyl-L-cysteine	15.3	-^b^	-
(±)-*S*-Methyl-L-cysteine sulfoxide	53.6	-	-
*S*-Ethyl-L-cysteine	29.7	-	-
L-Cysteine	1.6	-	-

^a ^The *V*_max _value was obtained by the rate of pyruvic acid production in the standard reaction mixture containing each substrate at 40 mM.

^b ^Not tested.

### Generation of a fungicidal activity in medium containing (±)-alliin and P-19486 alliinase

**Figure 5:**
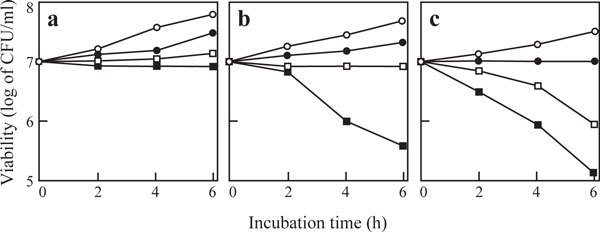
**Generation of a fungicidal activity in the presence of (±)-alliin and P-19486 alliinase**. *S. cerevisiae *cells were incubated in YPD medium containing (±)-alliin at 1 (a), 2 (b), and 4 mM (c), in which P-19486 alliinase wad supplemented at 0.04 (open circle), 0.16 (closed circle), 0.32 (open square), and 0.64 unit per ml (closed square). Viability was expressed as colony-forming units.

(±)-Alliin itself weakly inhibited the growth of *S. cerevisiae *cells at 40 mM (data not shown). In medium containing 1 mM (±)-alliin, however, the yeast cell growth was absolutely inhibited in the presence of P-19486 allinase at 0.64 unit per ml (Figure 5a). At the same dose, the enzyme could generate a significant lethal damage in medium containing (±)-alliin at 2 mM (Figure 5b). Under the condition, the rate of allicin production should be dependent on the substrate concentration, as deduced from the *K*m value of the enzyme (2.3 mM). As expected, the enzyme was effective for causing cell death at a lower dose (0.32 unit per ml) in medium where the concentration of (±)-alliin was increased up to 4 mM (Figure 5c). These results indicate the possibility of applying P-19486 alliinase for on-site generation of allicin-dependent fungicidal activity.

## Discussion

The alliinase-producing bacterium FERM P-19486 was identified as *E. adhaerens. E. adhaerens *constitutes a group of non-nodulating bacteria that do not harbor *nifH *gene, but the genus *Ensifer*, comprising the former *Sinorhizobium *species and *Ensifer adhaerens*, contain bacteria capable of nitrogen fixation in symbiosis with leguminous plants (Martens et al. 2008).

Plant alliinases so far reported are homodimeric glycoproteins consisting of two identical subunits (Schwimmer and Mazelis 1963; Mazelis and Crews 1968; Tobkin and Mazelis 1979; Nock and Mazelis 1986; Landshuter et al. 1994; Lohmüller et al. 1994; Rabinkov et al. 1994; Manabe et al. 1998; Kuettner 2002a, b). Microbial alliinase has been only roughly purified from a fungus *P. corymbiferum *so that general properties of the fungal enzyme are mostly kept unknown (Durbin and Uchytil 1971). P-19486 alliinase consisted of 2 homologous subunits and this homodimeric subunit composition is quite similar to those of the enzymes from garlic and onion. PLP is a cofactor essential for the C-S lyase activity of alliinase so far reported (Tobkin and Mazelis 1979; Kuettner 2002a, b; Shimon et al. 2007). Although each subunit of alliinase contains one tightly bound PLP, its exogenous addition enhances the enzyme activity as the purification proceeds (Schwimmer and Mazelis 1963; Mazelis and Crews 1968). Such an enhancement effect of PLP on the enzyme activity also suggested the involvement of bound PLP in the C-S lyase activity of P-19486 alliinase.

There exist two diastereomers, (+)-*S*-allyl-L-cysteine sulfoxide and (-)-*S*-allyl-L-cysteine sulfoxide, in the molecular structure of alliin because of the asymmetry of the sulfoxide group. These diastereomeric forms may not equally serve as a substrate for alliinase, if the stereochemically active center is involved in the enzyme reaction. Garlic alliinase more rapidly hydrolyzes (+)-alliin, a naturally occurring substrate for the enzymatic synthesis of allicin, than (-)-alliin (Stoll and Seeback 1951; Lancaster and Collin 1981; Kuettner et al. 2002b; Shimon et al. 2007). P-19486 alliinase seems to have a distinct amino acid sequence around the active site region for its strong contact with the sulfoxide group of (-)-alliin.

Alliinase shows a strict specificity toward alliin except that onion root isoforms, which are different in glycosylation, exhibit an additional activity toward L-cystine to a limited extent (Lancaster et al. 2000). Therefore, plant alliinase is fundamentally distinguished from *S*-alkyl L-cysteine lyase (EC 4.4.1.6), which stoichiometrically converts *S*-alkyl L-cysteine to *S*-alkyl mercaptan, pyruvic acid, and ammonia, as illustrated in Figure 1b (Schwimmer and Kjær 1960; Nomura et al. 1963; Mazelis and Creveling 1975). *S*-Alkylcysteine lyase from the bacterium *Pseudomonas cruciviae *similarly catalyzed the lyase action on (±)-*S*-alkyl-L-cysteine sulfoxide including (±)-alliin though the additional condensation reaction for allicin synthesis is not known for this bacterial enzyme (Nomura et al. 1963). In the sense, P-19486 alliinase is a novel enzyme that can alternatively exhibit the allicin synthetic activity on (-)-*S*-allyl-L-cysteine sulfoxide, (-)-alliin, and the C-S lyase activity on *S*-alkyl-L-cysteine (Table 3).

Allicin exhibits antifungal activity against various fungi including *S. cerevisiae *and the pathogenic yeast *Candia albicans *(Ankri and Mirelman 1999). In addition, allicin can enhance the fungicidal activity of amphotericin B, the most representative antifungal antibiotic, against these yeast strains (Ogita et al. 2006; Borjihan et al. 2009). A chemically synthesized allicin was used in these studies, whereas its enzymatic synthesis or even on-site production has been examined with the aid of garlic alliinase (Miron et al. 2003; Shadkchan et al. 2004; Fry et al. 2005; Slusarenko et al. 2008). It was doubtful whether P-19486 alliinase can be industrially or medically applied for allicin production because of its low thermal stability. However, this bacterial enzyme could effectively catalyze the corresponding allicin synthetic reaction using (±)-alliin at the concentration lower than the *K*m value (see Table 3 and Figure 4). In agreement with this fact, P-19486 alliinase could successfully generate a fungicidal activity using (±)-alliin as a precursor, which is easily synthesized by chemical oxidation of *S*-allyl-L-cysteine. The bacterial enzyme may be more beneficial when used in combination with garlic enzyme with respect to absolute conversion of (±)-alliin to allicin.

## Competing interests

The authors declare that they have no competing interests.

## Authors' contributions

MY and HT participated in all experiments except an assay of antifungal activity. HB carried out an assay of antifungal activity. AO, KF, and TT participated in design and coordination of this study. All authors read and approved the final manuscript.

## References

[B1] Agarwal KC Therapeutic actions of garlic constituents Med Res Rev 1996 16 111 124 10.1002/(SICI)1098-1128(199601)16:1<111::AID-MED4>3.0.CO;2-5 8788216

[B2] Ankri S Mirelman D Antimicrobial properties of allicin from garlic Microbes Infect 1999 1 125 129 10.1016/S1286-4579(99)80003-3 10594976

[B3] Block E The chemistry of garlic and onions Sci Am 1985 252 114 119 10.1038/scientificamerican0385-114 3975593

[B4] Borjihan H Ogita A Fujita K Hirasawa E Tanaka T The vacuole-targeting fungicidal activity of amphotericin B against the pathogenic fungus *Candida albicans *and its enhancement by allicin J Antibiot 2009 62 691 697 10.1038/ja.2009.103 19876074

[B5] Bradford MM A rapid and sensitive method for the quantitation of microgram quantities of protein utilizing the principle of protein-dye binding Anal Biochem 1976 72 248 254 10.1016/0003-2697(76)90527-3 942051

[B6] Casida LE Jr *Ensifer adhaerens *gen. nov., sp. nov.: a bacterial predator of bacteria in soil Int J Syst Bacteriol 1982 32 339 345 10.1099/00207713-32-3-339

[B7] Durbin RD Uchytil TF Purification and properties of alliin lyase from the fungus *Penicillium corymbiferum* Biochim Biophys Acta 1971 235 518 520

[B8] Fry FH Okarter N Baynton-Smith C Kershaw MJ Talbot NJ Jacob C Use of a substrate/alliinase combination to generate antifungal activity in situ J Agric Food Chem 2005 53 574 580 10.1021/jf048481j 15686404

[B9] Jones MG Hughes J Tregova A Milne J Tomestt AB Collin HA Biosynthesis of the flavor precursors of onion and garlic J Exp Botany 2004 55 1903 1918 10.1093/jxb/erh138 15234988

[B10] Kuettner EB Hilgenfeld R Weiss MS Purification, characterization, and crystallization of alliinase from garlic Arch Biochem Biophys 2002 402 192 200 10.1016/S0003-9861(02)00088-7 12051663

[B11] Kuettner EB Hilgenfeld R Weiss MS The active principle of garlic at atomic resolution J Biol Chem 2002 277 46402 46407 10.1074/jbc.M208669200 12235163

[B12] Lancaster JE Collin HA Presence of alliinase in isolated vacuoles and of alkyl cysteine sulphoxides in the cytoplasm of bulbs of onion (*Allium cepa*) Plant Sci Lett 1981 22 169 176 10.1016/0304-4211(81)90139-5

[B13] Lancaster JE Shaw ML Joyce MD McCallum JA McManus MT A novel alliinase from onion roots. Biochemical characterization and cDNA cloning Plant Physiol 2000 122 1269 1279 10.1104/pp.122.4.1269 10759524 PMC58963

[B14] Landshuter J Lohmüller EM Knobloch K Purification and characterization of a C-S-lyase from ramson, the wild garlic *Allium ursinum* Planta Med 1994 60 343 347 10.1055/s-2006-959497 17236056

[B15] Lohmüller EM Landshuter J Knobloch K On the isolation and characterization of a C-S-lyase preparation from leek, *Allium porrum* Planta Med 1994 60 337 342 10.1055/s-2006-959496 17236055

[B16] Manabe T Hasumi A Sugiyama M Yamazaki M Saito K Alliinase [*S*-alk(en)yl-L-cysteine sulfoxide lyase] from *Allium tuberosum *(Chinese chive) - purification, localization, cDNA cloning and heterologous functional expression Eur J Biochem 1998 257 21 30 10.1046/j.1432-1327.1998.2570021.x 9799098

[B17] Martens M Dawyndt P Coopman R Gillis M De Vos P Willems A Advantages of multilocus sequence analysis for taxonomic studies: a case study using 10 housekeeping genes in the genus *Ensifer *(including former *Sinorhizobium*) Int J Syst Evol Microbiol 2008 58 200 214 10.1099/ijs.0.65392-0 18175710

[B18] Mazekis M Creveling RK Purification and properties of *S*-alkyl-L-cysteine lyase from seedlings of *Acacia farnesiana *Willd Biochem J 1975 147 485 491 241329 10.1042/bj1470485 PMC1165476

[B19] Mazelis M Crews L Purification of the alliin lyase of garlic, *Allium sativum *L Biochem J 1968 108 725 730 4970593 10.1042/bj1080725 PMC1198877

[B20] Miron T Mironchik M Mirelman D Wilchek M Rabinkov A Inhibition of tumor growth by a novel approach: in situ allicin generation using targeted alliinase delivery Mol Cancer Ther 2003 2 1295 1301 14707270

[B21] Murakami F Studies on the nutritional value of *Allium *plants: (XXXVII) Decomposition of alliin homologues by acetone-powdered enzyme preparation of *Bacillus subtilis* Vitamins 1960 20 131 135

[B22] Nock LP Mazelis M The C-S lyases of higher plants: preparation and properties of homogeneous alliin lyase from garlic (*Allium sativum*) Arch Biochem Biophys 1986 249 27 33 10.1016/0003-9861(86)90556-4 3740854

[B23] Nomura J Nishizuka Y Hayaishi O *S*-Alkylcysteinase: Enzymatic cleavage of *S*-methyl-L-cysteine and its sulfoxide J Biol Chem 1963 238 1441 1446

[B24] Ogita A Fujita K Taniguchi M Tanaka T Enhancement of the fungicidal activity of amphotericin B by allicin, an allyl-sulfur compound from garlic, against the yeast *Saccharomyces cerevisiae *as a model system Planta Med 2006 72 1247 1250 10.1055/s-2006-947203 16902870

[B25] Rabinkov A Zhu XZ Grafi G Galili G Mirelman D Alliin lyase (alliinase) from garlic (*Allium sativum*). Biochemical characterization and cDNA cloning Appl Biochem Biotchnol 1994 48 149 171 10.1007/BF02788739 7979352

[B26] Rogel MA Hernández-Lucas I Kuykendall LD Balkwill DL Martinez-Romero E Nitrogen-fixing nodules with *Ensifer adhaerens *harboring *Rhizobium tropici *symbiotic plasmids Appl Environ Microbiol 2001 67 3264 3268 10.1128/AEM.67.7.3264-3268.2001 11425750 PMC93009

[B27] Sawada H Kuykendall LD Young JM Changing concepts in the systematics of bacterial nitrogen-fixing legume symbionts J Gen Appl Microbiol 2003 49 155 179 10.2323/jgam.49.155 12949698

[B28] Schwimmer S Kjær A Purification and specificity of the C-S-lyase of *Albizzia lophanta* Biochim Biophys Acta 1960 42 316 324 10.1016/0006-3002(60)90795-2 13749270

[B29] Schwimmer S Mazelis M Characterization of alliinase of *Allium cepa *(onion) Arch Biochem Biophys 1963 100 66 73 10.1016/0003-9861(63)90035-3 13987494

[B30] Shadkchan Y Shemesh E Mirelman D Miron T Rabinkov A Wilchek M Osherov N Efficacy of allicin, the reactive molecule of garlic, in inhibiting *Aspergillus *spp. *in vitro*, and in a murine model of disseminated aspergillosis J Antimicrob Chemother 2004 53 832 836 10.1093/jac/dkh174 15044429

[B31] Shimon JWL Rabinkov A Shin I Miron T Mirelman D Wilchek M Frolow F Two structures of alliinase from *Alliium sativum *L.: Apo form and ternary complex with aminoacrylate reaction intermediate covalently bound to the PLP cofactor J Mol Biol 2007 366 611 625 10.1016/j.jmb.2006.11.041 17174334

[B32] Siegel G Walter A Engel S Walper A Michel F Pleiotropic effects of garlic Wien Med Wochenschr 1999 149 217 224 10483684

[B33] Slusarenko AJ Patel A Portz D Control of plant disease by natural products: allicin from garlic as a case study Eur J Plant Pathol 2008 121 313 322 10.1007/s10658-007-9232-7

[B34] Stoll A Seeback E Chemical investigation on alliin, the specific principle of garlic Adv Enzymol 1951 11 337 400 10.1002/9780470122563.ch8 24540596

[B35] Tobkin HE Jr Mazelis M Alliin lyase: preparation and characterization of the homogeneous enzyme from onion bulbs Arch Biochem Biophys 1979 193 150 157 10.1016/0003-9861(79)90018-3 453844

[B36] Tsai Y Cole LL Davis LE Lockwood SJ Simmons V Wild GC Antiviral properties of garlic: in vitro effects on influenza B, herpes simplex and coxsackie viruses Planta Med 1985 51 460 461 10.1055/s-2007-969553 3001801

